# A rare case report of supernumerary nostril in an infant: clinical presentation, surgical management, and literature review

**DOI:** 10.1097/MS9.0000000000004262

**Published:** 2025-11-04

**Authors:** Dipesh Kumar Singh, Nitesh Pandit, Isha Dhakal, Ajay Kumar Yadav

**Affiliations:** B.P. Koirala Institute of Health Sciences, Dharan, Nepal

**Keywords:** accessory nasal tract, case report, congenital anomaly, nasal development, pediatric plastic surgery, supernumerary nostril

## Abstract

**Introduction and Importance:**

Supernumerary nostril is an exceedingly rare congenital malformation characterized by the presence of an accessory nostril in addition to the normal nasal openings. The anomaly may or may not communicate with the nasal cavity.

**Case Presentation:**

We report a case of a 7-month-old female infant presenting with a tubular structure superior to the left nasal ala, consistent with a supernumerary nostril. Contrast-enhanced computed tomography (CT) of the face demonstrated a blind-ending soft-tissue tubular tract without communication to the nasal cavity or paranasal sinuses. There were no encephaloceles, bone deformities, or other midline anomalies identified. The tract was completely excised, and the left nasal ala was reconstructed with layered primary closure. Histopathology confirmed ciliated respiratory epithelium lining the tract. Postoperative recovery was uneventful. At 12-month follow-up, the patient demonstrated satisfactory cosmetic symmetry and no evidence of recurrence.

**Discussion:**

Early recognition and surgical management of supernumerary nostrils are important to optimize functional and aesthetic outcomes. Detailed preoperative imaging is valuable to exclude intracranial or intranasal communication and to plan reconstruction.

**Conclusion:**

Given its rarity, each reported case enriches current understanding of clinical variability and management strategies.

## Introduction

Congenital anomalies of the external nose are rare entities, often associated with other craniofacial malformations. Among these, the supernumerary nostril represents a unique and exceedingly rare developmental anomaly characterized by the presence of an additional nostril-like structure, either communicating with the nasal cavity or existing independently. Since the first description by Lindsay in 1906, approximately 60 cases have been reported in the literature^[1]^. The embryological mechanism is not fully understood but is thought to involve aberrant development of the lateral nasal processes or an accessory nasal placode during early embryogenesis (third to fourth weeks of gestation)^[2]^.

Supernumerary nostrils are typically present at birth as a cosmetic deformity and may cause psychological distress. Rarely, they may result in functional impairment if associated with airway obstruction or recurrent infections. Early diagnosis using clinical assessment supplemented by imaging is crucial to delineate the extent and to rule out associated structural defects. Surgical excision is the treatment of choice aimed at removing the accessory tract and restoring nasal contour while minimizing scarring. This report details an isolated left-sided supernumerary nostril in an infant, managed at a tertiary academic center, and includes a concise review of relevant contemporary literature.

**HIGHLIGHTS**

- Rare Congenital Anomaly: Supernumerary nostril is an extremely rare congenital condition, with approximately 60 cases reported globally, typically presenting as a unilateral accessory nostril.
- Case Overview: A 7-month-old female infant presented with a blind-ending, tubular accessory nostril above the left nasal ala, without communication with the nasal cavity or associated anomalies.
- Imaging and Diagnosis: CT imaging confirmed a noncommunicating soft-tissue tract with no intracranial or bony involvement, aiding in precise surgical planning.
- Surgical Management: Complete excision of the accessory tract was performed under general anesthesia, followed by cosmetic reconstruction of the nasal ala, resulting in excellent postoperative outcomes.
- Clinical Significance: Early recognition and surgical correction are essential for optimal aesthetic and functional outcomes, and case reporting contributes valuable data to the limited literature on this anomaly.

## Methods

Study design: The study is a case report (prospective, unicenter, formal, consecutive, clinical). The work has been reported in line with the SCARE 2025 criteria^[3]^.

## Case presentation

A 7-month-old female infant was brought by her parents with a tubular, skin-covered structure noted since birth superior to the left nasal ala. There was no history of respiratory distress, feeding difficulty, or recurrent infections. Antenatal history was unremarkable, and there was no family history of craniofacial anomalies. Growth and developmental milestones were appropriate for age.

**Figure 1.**
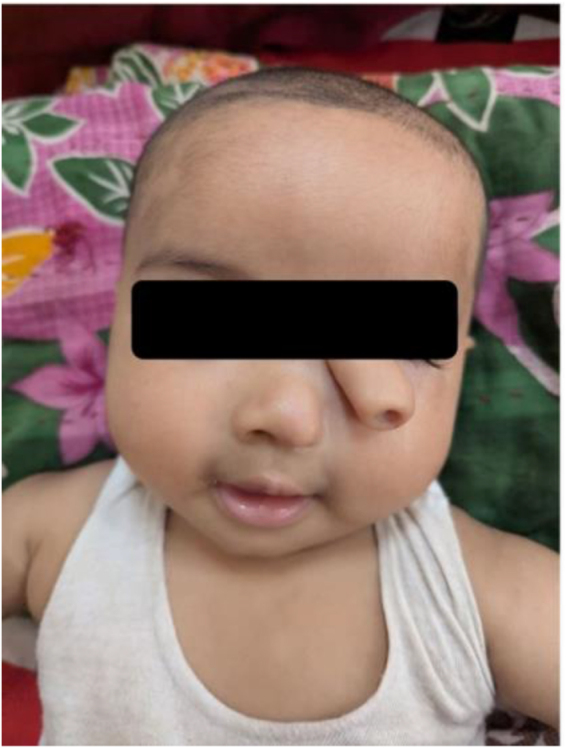
Clinical photograph showing a 7-month-old infant with a tubular accessory structure superior to the left nasal ala, consistent with a supernumerary nostril. The orifice was patent but did not communicate with the underlying nasal cavity.

On examination, a 1.0–1.5 cm tubular tract with a small distal orifice was present superior to the left nostril, directed anteromedially toward the medial canthal region (Fig. 1). Gentle probing in the clinic was nonpenetrating and produced no nasal discharge. External nasal symmetry was otherwise preserved, and no other craniofacial anomalies were identified on clinical inspection.

Contrast-enhanced computed tomography (CT) of the face demonstrated a well-defined, soft-tissue tubular tract in the left nasal ala region, which terminated blindly with no communication to the nasal cavity or paranasal sinuses. No bony defect, encephalocele, or intracranial extension was identified. These imaging findings guided the surgical plan for complete local excision of the tract.

Following informed written parental consent, the infant underwent surgery under general anesthesia. A circumferential skin incision was made around the tract, and meticulous dissection carried the tract to its blind end. The tract was excised en bloc. Hemostasis was secured, and layered primary closure was performed to reconstitute the left nasal ala contours. No intraoperative complications occurred. The specimen was sent for histopathological examination, which revealed ciliated respiratory mucosa consistent with accessory nasal tract tissue.

The postoperative course was unremarkable. Wound healing was uneventful, without infection or dehiscence. Postoperative follow-up included clinical review at 1 week, 1 month, 3 months, and 12 months. Clinician-assessed outcomes demonstrated satisfactory nasal contour at 1 and 3 months, with maintained symmetry, absence of hypertrophic scarring, and no clinical or parental concerns regarding nasal function at 12 months.

The patient was managed at a tertiary academic hospital within a multidisciplinary framework involving pediatric surgery, plastic/reconstructive surgery, and pediatric anesthesia. Standard perioperative protocols at our center included preoperative imaging with contrast-enhanced CT for craniofacial tract delineation, antibiotic prophylaxis per pediatric guidelines, intraoperative loupe magnification for careful tract excision, and layered primary closure with attention to nasal subunits. Parent-reported outcomes, obtained via informal structured interview, indicated age-appropriate feeding and no airway symptoms at each follow-up time point.

## Discussion

A supernumerary nostril represents a rare congenital anomaly of the nasal structure. Normal embryogenesis of the nose involves the coordinated fusion of the frontonasal, medial nasal, and lateral nasal processes between the 3rd and 10th weeks of gestation. Any disruption, duplication, or abnormal budding during this critical period can result in nasal anomalies, including supernumerary nostrils^[4]^.

The pathogenesis remains unclear. One widely accepted theory suggests that an accessory nasal placode may develop, leading to an additional invagination and formation of an accessory nostril. Another hypothesis proposes splitting or duplication of the lateral nasal process^[5]^.

Clinically, supernumerary nostrils are usually unilateral, with a slight left-sided predominance^[1]^. The accessory nostril may be located medial, lateral, or superior to the normal nostril, and communication with the nasal cavity is variable. Rarely, bilateral cases have been described, sometimes associated with syndromic craniofacial malformations such as facial clefts or hypertelorism^[6]^.

Differential diagnoses include nasal dermoid cysts, nasal encephaloceles, and frontonasal dysplasia, necessitating thorough clinical and radiological assessment. CT imaging is essential for delineating soft tissue and bony involvement, planning surgical excision, and ruling out deeper communications.

Management aims for complete surgical excision of the accessory tract, restoration of nasal contour, and prevention of functional and psychological sequelae. Early intervention is advocated to minimize scarring and support normal facial development. Surgical planning was considered an individualized technique depending on the tract’s complexity, size, and relation to adjacent structures.

The outcome largely depends on the complexity of the anomaly and associated malformations^[7]^. Isolated cases, such as the one presented, generally have an excellent prognosis following appropriate surgical management. Postoperative follow-up at 1 week, 1 month, 3 months, and 12 months demonstrated stable cosmetic results with no recurrence or functional impairment^[8]^. Longer-term follow-up is recommended to assess growth-related changes and potential late complications^[9]^.

In our case, the accessory nostril was isolated without any associated anomalies. Surgical excision and primary reconstruction provided satisfactory cosmetic results without functional impairment, aligning with outcomes reported in the limited existing literature.

Limitations of this report include its single-case nature, which limits generalizability, and the relatively short follow-up. Nevertheless, detailed documentation of clinical, radiological, and surgical findings contributes to the limited literature on this anomaly. Further multicenter studies and cumulative case reports are needed to refine surgical protocols and better understand long-term outcomes.

## Conclusion

Supernumerary nostril is a rare congenital anomaly that may be isolated or associated with other craniofacial abnormalities, which pose both cosmetic and functional challenges. A high index of suspicion, early recognition, appropriate imaging to exclude intranasal or intracranial communication, and complete surgical excision with attention to reconstructive principles typically yield favorable functional and cosmetic outcomes. Reporting such rare presentations adds to the growing body of knowledge and assists clinicians in recognizing and managing this unique condition.

## Data Availability

The data that support the findings of this study are available from the corresponding author upon reasonable request.
